# (1*R*,4*S*)-7,8-Dichloro-1,2,3,4-tetra­hydro-1,11,11-trimethyl-1,4-methano­phenazine

**DOI:** 10.1107/S1600536810044016

**Published:** 2010-10-31

**Authors:** Guy Crundwell, Neil Glagovich

**Affiliations:** a Department of Chemistry and Biochemistry, Central Connecticut State University, 1619 Stanley Street, New Britain, CT 06053, USA

## Abstract

The title compound, C_16_H_16_Cl_2_N_2_, was synthesized by the condensation reaction between 4,5-dichloro-*o*-phenyl­ene­diamine and (1*R*)-(-)-camphorquinone in boiling acetic acid. The two crystallographically independent mol­ecules in the unit cell are related by a pseudo-inversion center.

## Related literature

Steel & Fitchett (2000 ▶, 2006 ▶) illustrate the use of stereochemically active quinoxalines in extended metal–ligand networks.
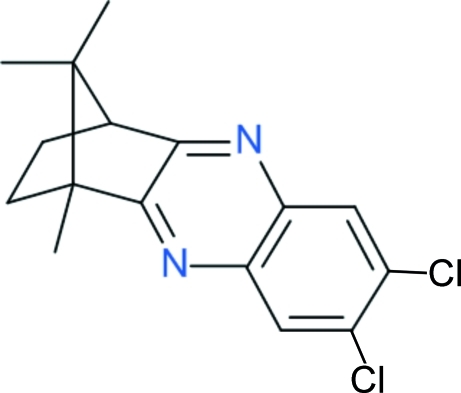


## Experimental

### 

#### Crystal data

C_16_H_16_Cl_2_N_2_
*M* *_r_* = 307.21
Monoclinic, 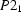

*a* = 6.9741 (3) Å
*b* = 13.0892 (5) Å
*c* = 16.9594 (5) Å
β = 101.701 (3)°
*V* = 1515.97 (10) Å^3^
*Z* = 4
Mo *K*α radiation
μ = 0.42 mm^−1^
*T* = 293 K
0.32 × 0.18 × 0.11 mm

#### Data collection

Oxford Xcalibur Sapphire3 diffractometer
Absorption correction: multi-scan (*CrysAlis PRO*; Oxford Diffraction, 2009 ▶) *T*_min_ = 0.897, *T*_max_ = 1.000
42674 measured reflections
12344 independent reflections
7343 reflections with *I* > 2σ(*I*)
*R*_int_ = 0.032

#### Refinement

*R*[*F* ^2^ > 2σ(*F* ^2^)] = 0.058
*wR*(*F* ^2^) = 0.163
*S* = 0.93
12344 reflections
367 parameters
1 restraint
H-atom parameters constrained
Δρ_max_ = 0.42 e Å^−3^
Δρ_min_ = −0.18 e Å^−3^
Absolute structure: Flack (1983 ▶), with 5825 Friedel pairs
Flack parameter: 0.03 (5)

### 

Data collection: *CrysAlis CCD* (Oxford Diffraction, 2009 ▶); cell refinement: *CrysAlis CCD*; data reduction: *CrysAlis RED* (Oxford Diffraction, 2009 ▶); program(s) used to solve structure: *SHELXS97* (Sheldrick, 2008 ▶); program(s) used to refine structure: *SHELXL97* (Sheldrick, 2008 ▶); molecular graphics: *ORTEP-3* (Farrugia, 1997 ▶), *Mercury* (Macrae *et al.*, 2008 ▶) and *PLATON* (Spek, 2009 ▶); software used to prepare material for publication: *SHELXTL* (Sheldrick, 2008 ▶).

## Supplementary Material

Crystal structure: contains datablocks I, global. DOI: 10.1107/S1600536810044016/zl2314sup1.cif

Structure factors: contains datablocks I. DOI: 10.1107/S1600536810044016/zl2314Isup2.hkl

Additional supplementary materials:  crystallographic information; 3D view; checkCIF report

## Figures and Tables

**Fig. 1.**
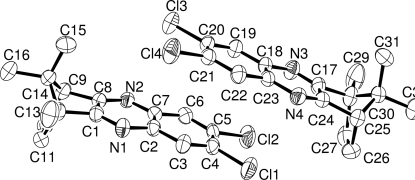
A view of (1R,4S)-7,8-Dichloro-1,2,3,4-tetrahydro-1,11,11- trimethyl-1,4-methanophenazine (Farrugia, 1997). There are two molecules in the asymmetric unit. Displacement ellipsoids are drawn at the 50% probability level. Labels for atoms C10 and C12 in the first molecule and atom C28 in the second molecule were omitted for clarity.

**Fig. 2.**
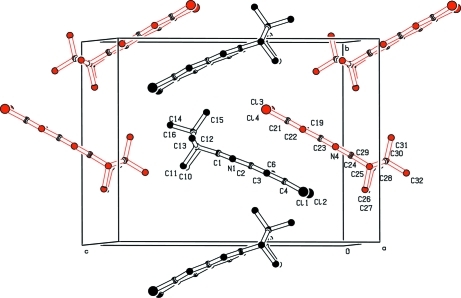
A view of the packing nearly along (100) showing both molecules as well as the slight quinoxaline plane bending in the first (Spek, 2009).

**Fig. 3.**
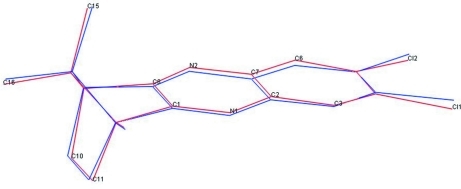
Molecular overlay of both molecules in the asymmetric unit showing the slight bend of molecule 1 [red] compared to molecule 2 [blue] (Macrae et al., 2008).

## supplementary crystallographic information

### Comment

Nitrogen-containing aromatic heterocycles have often been used as ligands in one-, two-, and three dimensional metal–organic coordination polymers. There has been interest in developing chiral nitrogen-containing aromatic heterocycles in order to have greater design control over the assembly of these extended networks in the solid state (Steel & Fitchett, 2000). As a subset of nitrogen-containing aromatic heterocycles, quinoxalines, pyrazino[2,3-*g*]quinoxalines, and phenazines have shown the ability to bind to a variety of metals and are, as ligands, easy to synthesize *via* condensation reactions between ethanediones/quinones and diamines/tetraamines (Steel & Fitchett, 2006). In this paper we report the synthesis and structure of the chiral (1*R*,4S)-7,8-dichloro-1,2,3,4-tetrahydro-1,11,11-trimethyl-1,4-methanophenazine.

The title compound crystallizes in a chiral setting in the space group P2_1_ with two crystallographically independent molecules in the asymmetric unit, Fig. 1. The two molecules are closely related by a pseudo inversion center located near coordinates x = 0.263, y = 0.461, z = 0.252. All bond distances and angles fall within expected values and there are no classic hydrogen bonds; however as can be seen in Fig. 2, one of the molecules packs with a slight bend in the quinoxaline moiety. Fig. 3 shows the molecular overlay of the two molecules in the asymmetric unit.

### Experimental

To a 150 ml round bottom flask equipped with a reflux condenser was added 2.9 g (0.0120 mol) (1*R*)-(-)-camphorquinone, 2.77 g (0.0156 mol) 4,5-dichloro-*o*-phenylenediamine, and 50 ml glacial acetic acid. The mixture was heated to reflux for 3 h, and was then poured over ice to precipitate the crude product. After isolation *via* vacuum filtration, the crude product was recrystallized from methanol to yield 2.79 g (0.00908 mol) 3,4-dichlorocamphorquinoxaline (75% yield).

MP (K): 422.3-424.0; IR (CHCl_3_): 3086, 2051, 1521, 1404, 12635, 1166, 1118, 890, 876 cm^-1^; ^1^H NMR (300 MHz, CDCl_3_): δ 8.13 (*s*, 1H), 8.06 (*s*, 1H), 3.04 (*d*, 1H, *J* = 4.6 Hz), 2.31 (*dtd*, 1H, *J* = 4.6 Hz, *J* = 8 Hz, *J* = 12 Hz), 2.06 (*dq*, 1H, *J* = 8 Hz, *J* = 12 Hz), 1.40 (*s*, 3H), 1.39 (*q*, 2H, *J* = 10 Hz), 1.11 (*s*, 3H), 0.60 (*s*, 3H); ^13^C NMR (300 MHz, CDCl_3_): δ 166.8, 165.0, 140.4, 140.3, 132.2, 129.7, 129.5, 54.3, 54.0, 53.3, 31.8, 24.6, 20.4, 18.5, 10.0; UV/Vis (CH_2_Cl_2_; λ_max_) 260, 267, 365; MS (calculated for C_16_H_16_Cl_2_N_2_): *M*^+^: 306, measured: 306.

### Refinement

H atoms were included in calculated positions with C—H distances of 0.93 Å, 0.96 Å, 0.97 Å, and 0.98 Å based upon type of carbon and were included in the refinement in riding motion approximation with *U*_iso_ = 1.2*U*_eq_ of the carrier atom.

### Crystal data

**Table:**

C_16_H_16_Cl_2_N_2_	*F*(000) = 640
*M**_r_* = 307.21	*D*_x_ = 1.346 Mg m^−^^3^
Monoclinic, *P*2_1_	Melting point: 422 K
Hall symbol: P 2yb	Mo *K*α radiation, λ = 0.71073 Å
*a* = 6.9741 (3) Å	Cell parameters from 15315 reflections
*b* = 13.0892 (5) Å	θ = 4.2–35.0°
*c* = 16.9594 (5) Å	µ = 0.42 mm^−^^1^
β = 101.701 (3)°	*T* = 293 K
*V* = 1515.97 (10) Å^3^	Block, white
*Z* = 4	0.32 × 0.18 × 0.11 mm

### Data collection

**Table:**

Oxford Xcalibur Sapphire3 diffractometer	12344 independent reflections
Radiation source: Enhance (Mo) X-ray Source	7343 reflections with *I* > 2σ(*I*)
graphite	*R*_int_ = 0.032
Detector resolution: 16.1790 pixels mm^-1^	θ_max_ = 35.0°, θ_min_ = 4.2°
ω scans	*h* = −11→11
Absorption correction: multi-scan (*CrysAlis PRO*; Oxford Diffraction, 2009)	*k* = −20→20
*T*_min_ = 0.897, *T*_max_ = 1.000	*l* = −27→27
42674 measured reflections	

### Refinement

**Table:**

Refinement on *F*^2^	Secondary atom site location: difference Fourier map
Least-squares matrix: full	Hydrogen site location: inferred from neighbouring sites
*R*[*F*^2^ > 2σ(*F*^2^)] = 0.058	H-atom parameters constrained
*wR*(*F*^2^) = 0.163	*w* = 1/[σ^2^(*F*_o_^2^) + (0.0946*P*)^2^ + 0.0917*P*] where *P* = (*F*_o_^2^ + 2*F*_c_^2^)/3
*S* = 0.93	(Δ/σ)_max_ < 0.001
12344 reflections	Δρ_max_ = 0.42 e Å^−^^3^
367 parameters	Δρ_min_ = −0.18 e Å^−^^3^
1 restraint	Absolute structure: Flack (1983), with 5825 Friedel pairs
Primary atom site location: structure-invariant direct methods	Flack parameter: 0.03 (5)

### Special details

**Table:**

Experimental. Absorption correction: CrysAlis Pro (Oxford Diffraction Ltd., 2009) Empirical absorption correction using spherical harmonics, implemented in SCALE3 ABSPACK scaling algorithm.Hydrogen atoms were included in calculated positions with a C—H distances of 0.93 Å, 0.96 Å, 0.97 Å, and 0.98 Å based upon type of carbon. Hydrogen atoms were included in the refinement in riding motion approximation with a *U*_iso_ of either 1.2*U*_eq_ or 1.5*U*_eq_ of the carrier atom depending upon type of carbon.
Geometry. All e.s.d.'s (except the e.s.d. in the dihedral angle between two l.s. planes) are estimated using the full covariance matrix. The cell e.s.d.'s are taken into account individually in the estimation of e.s.d.'s in distances, angles and torsion angles; correlations between e.s.d.'s in cell parameters are only used when they are defined by crystal symmetry. An approximate (isotropic) treatment of cell e.s.d.'s is used for estimating e.s.d.'s involving l.s. planes.
Refinement. Refinement of *F*^2^ against ALL reflections. The weighted *R*-factor *wR* and goodness of fit *S* are based on *F*^2^, conventional *R*-factors *R* are based on *F*, with *F* set to zero for negative *F*^2^. The threshold expression of *F*^2^ > σ(*F*^2^) is used only for calculating *R*-factors(gt) *etc*. and is not relevant to the choice of reflections for refinement. *R*-factors based on *F*^2^ are statistically about twice as large as those based on *F*, and *R*-factors based on ALL data will be even larger.

### Fractional atomic coordinates and isotropic or equivalent isotropic displacement parameters (Å^2^)

**Table:**

	*x*	*y*	*z*	*U*_iso_*/*U*_eq_	
C1	0.1545 (3)	0.45050 (16)	0.49934 (12)	0.0398 (4)	
N1	0.2970 (3)	0.42161 (16)	0.46481 (11)	0.0460 (4)	
C2	0.2335 (3)	0.38885 (17)	0.38582 (12)	0.0402 (4)	
C3	0.3738 (3)	0.35025 (19)	0.34450 (14)	0.0465 (5)	
H3	0.5062	0.3515	0.3684	0.056*	
C4	0.3134 (4)	0.31076 (17)	0.26865 (14)	0.0442 (5)	
Cl1	0.48375 (11)	0.25649 (6)	0.22037 (4)	0.0677 (2)	
C5	0.1167 (4)	0.31228 (18)	0.23080 (12)	0.0451 (5)	
Cl2	0.03974 (13)	0.25733 (7)	0.13693 (4)	0.0764 (2)	
C6	−0.0217 (4)	0.35345 (19)	0.26854 (13)	0.0471 (5)	
H6	−0.1519	0.3572	0.2418	0.057*	
C7	0.0347 (3)	0.39014 (16)	0.34825 (12)	0.0382 (4)	
N2	−0.1129 (3)	0.42215 (15)	0.38665 (11)	0.0423 (4)	
C8	−0.0476 (3)	0.45064 (16)	0.46059 (12)	0.0393 (4)	
C9	−0.1600 (4)	0.48131 (19)	0.52398 (14)	0.0467 (5)	
H9	−0.2939	0.5059	0.5043	0.056*	
C10	−0.1369 (4)	0.38915 (18)	0.58179 (15)	0.0521 (5)	
H10A	−0.1703	0.3259	0.5524	0.063*	
H10B	−0.2196	0.3966	0.6211	0.063*	
C11	0.0802 (4)	0.39084 (19)	0.62275 (14)	0.0534 (6)	
H11A	0.1447	0.3281	0.6122	0.064*	
H11B	0.0956	0.3994	0.6805	0.064*	
C12	0.1656 (4)	0.48392 (19)	0.58474 (13)	0.0467 (5)	
C13	0.3638 (5)	0.5211 (3)	0.62876 (18)	0.0753 (9)	
H13A	0.4578	0.4670	0.6314	0.113*	
H13B	0.3556	0.5416	0.6823	0.113*	
H13C	0.4038	0.5782	0.6005	0.113*	
C14	−0.0131 (4)	0.55950 (18)	0.57193 (13)	0.0489 (5)	
C15	0.0151 (6)	0.6545 (2)	0.52444 (19)	0.0738 (9)	
H15A	0.0376	0.6351	0.4725	0.111*	
H15B	0.1258	0.6923	0.5529	0.111*	
H15C	−0.1001	0.6963	0.5179	0.111*	
C16	−0.0675 (5)	0.5936 (2)	0.65206 (17)	0.0650 (7)	
H16A	0.0376	0.6334	0.6826	0.097*	
H16B	−0.0892	0.5344	0.6825	0.097*	
H16C	−0.1846	0.6341	0.6407	0.097*	
C17	0.3327 (3)	0.43610 (18)	0.00994 (12)	0.0440 (5)	
N3	0.1985 (3)	0.47672 (17)	0.04292 (11)	0.0496 (5)	
C18	0.2727 (3)	0.52064 (17)	0.11783 (12)	0.0396 (4)	
C19	0.1397 (3)	0.56594 (19)	0.15972 (13)	0.0459 (5)	
H19	0.0066	0.5669	0.1370	0.055*	
C20	0.2070 (3)	0.60867 (17)	0.23410 (13)	0.0426 (5)	
Cl3	0.04203 (10)	0.66515 (6)	0.28452 (4)	0.06479 (19)	
C21	0.4066 (4)	0.60690 (17)	0.26942 (13)	0.0445 (5)	
Cl4	0.49046 (12)	0.66082 (7)	0.36273 (4)	0.0725 (2)	
C22	0.5387 (4)	0.56247 (18)	0.22961 (13)	0.0456 (5)	
H22	0.6712	0.5610	0.2534	0.055*	
C23	0.4732 (3)	0.51941 (16)	0.15314 (12)	0.0391 (4)	
N4	0.6126 (3)	0.47573 (15)	0.11472 (11)	0.0463 (4)	
C24	0.5377 (3)	0.43543 (16)	0.04548 (12)	0.0413 (4)	
C25	0.6353 (4)	0.37835 (19)	−0.01252 (13)	0.0498 (5)	
H25	0.7758	0.3907	−0.0074	0.060*	
C26	0.5749 (5)	0.2668 (2)	−0.00338 (16)	0.0633 (7)	
H26A	0.6464	0.2209	−0.0319	0.076*	
H26B	0.5978	0.2470	0.0529	0.076*	
C27	0.3554 (5)	0.2660 (2)	−0.04104 (17)	0.0693 (8)	
H27A	0.2791	0.2455	−0.0018	0.083*	
H27B	0.3277	0.2197	−0.0866	0.083*	
C28	0.3087 (4)	0.3782 (2)	−0.06852 (13)	0.0540 (6)	
C29	0.1155 (5)	0.3946 (4)	−0.12606 (19)	0.0893 (12)	
H29A	0.0101	0.3769	−0.0999	0.134*	
H29B	0.1097	0.3523	−0.1727	0.134*	
H29C	0.1037	0.4650	−0.1421	0.134*	
C30	0.5031 (4)	0.40956 (18)	−0.09395 (13)	0.0495 (5)	
C31	0.5182 (6)	0.5232 (2)	−0.11237 (17)	0.0722 (9)	
H31A	0.6463	0.5377	−0.1221	0.108*	
H31B	0.4964	0.5627	−0.0673	0.108*	
H31C	0.4214	0.5405	−0.1592	0.108*	
C32	0.5429 (5)	0.3497 (2)	−0.16611 (15)	0.0638 (7)	
H32A	0.4404	0.3628	−0.2121	0.096*	
H32B	0.5470	0.2780	−0.1540	0.096*	
H32C	0.6662	0.3706	−0.1777	0.096*	

### Atomic displacement parameters (Å^2^)

**Table:**

	*U*^11^	*U*^22^	*U*^33^	*U*^12^	*U*^13^	*U*^23^
C1	0.0469 (11)	0.0376 (10)	0.0361 (9)	−0.0027 (8)	0.0115 (8)	−0.0006 (7)
N1	0.0440 (10)	0.0562 (11)	0.0383 (8)	−0.0062 (8)	0.0098 (7)	−0.0093 (8)
C2	0.0465 (11)	0.0411 (10)	0.0354 (9)	−0.0067 (9)	0.0142 (8)	−0.0013 (8)
C3	0.0435 (12)	0.0503 (13)	0.0499 (12)	−0.0078 (10)	0.0193 (9)	−0.0088 (10)
C4	0.0569 (13)	0.0377 (10)	0.0449 (11)	−0.0050 (9)	0.0270 (10)	0.0006 (9)
Cl1	0.0768 (4)	0.0686 (4)	0.0686 (4)	−0.0012 (3)	0.0402 (3)	−0.0185 (4)
C5	0.0673 (14)	0.0421 (11)	0.0277 (9)	−0.0043 (10)	0.0141 (9)	0.0011 (8)
Cl2	0.0963 (5)	0.0933 (5)	0.0377 (3)	0.0067 (5)	0.0092 (3)	−0.0173 (3)
C6	0.0516 (13)	0.0508 (13)	0.0364 (10)	0.0011 (10)	0.0028 (9)	0.0022 (9)
C7	0.0448 (11)	0.0364 (10)	0.0349 (9)	0.0002 (8)	0.0119 (8)	0.0046 (8)
N2	0.0438 (10)	0.0466 (10)	0.0364 (8)	0.0065 (8)	0.0076 (7)	−0.0009 (7)
C8	0.0435 (11)	0.0374 (10)	0.0390 (9)	0.0035 (8)	0.0132 (8)	0.0026 (8)
C9	0.0513 (12)	0.0463 (11)	0.0464 (11)	0.0062 (9)	0.0193 (9)	−0.0040 (9)
C10	0.0673 (15)	0.0422 (11)	0.0545 (12)	−0.0003 (10)	0.0304 (11)	−0.0004 (10)
C11	0.0784 (17)	0.0469 (12)	0.0384 (10)	0.0112 (11)	0.0205 (11)	0.0076 (9)
C12	0.0568 (13)	0.0491 (12)	0.0354 (9)	−0.0005 (10)	0.0118 (9)	−0.0071 (9)
C13	0.0713 (19)	0.102 (2)	0.0514 (15)	−0.0163 (17)	0.0087 (13)	−0.0273 (16)
C14	0.0688 (14)	0.0381 (11)	0.0431 (10)	0.0026 (10)	0.0185 (10)	−0.0035 (9)
C15	0.122 (3)	0.0364 (13)	0.0670 (17)	−0.0035 (16)	0.0291 (18)	0.0021 (12)
C16	0.091 (2)	0.0517 (14)	0.0576 (14)	0.0095 (14)	0.0271 (14)	−0.0129 (12)
C17	0.0555 (12)	0.0448 (11)	0.0320 (9)	−0.0028 (10)	0.0094 (8)	−0.0052 (8)
N3	0.0462 (11)	0.0618 (12)	0.0403 (9)	0.0016 (9)	0.0078 (8)	−0.0121 (9)
C18	0.0440 (11)	0.0406 (10)	0.0345 (9)	−0.0013 (8)	0.0088 (8)	−0.0049 (8)
C19	0.0445 (12)	0.0550 (13)	0.0389 (10)	0.0013 (10)	0.0104 (9)	−0.0075 (10)
C20	0.0554 (12)	0.0366 (10)	0.0391 (10)	−0.0022 (9)	0.0174 (9)	−0.0054 (8)
Cl3	0.0682 (4)	0.0729 (4)	0.0587 (4)	0.0040 (3)	0.0257 (3)	−0.0225 (3)
C21	0.0595 (13)	0.0402 (11)	0.0344 (9)	−0.0056 (10)	0.0108 (9)	−0.0050 (8)
Cl4	0.0801 (4)	0.0897 (5)	0.0448 (3)	−0.0031 (4)	0.0059 (3)	−0.0286 (3)
C22	0.0523 (13)	0.0483 (12)	0.0360 (10)	−0.0011 (10)	0.0083 (9)	−0.0055 (9)
C23	0.0496 (12)	0.0366 (10)	0.0316 (9)	−0.0007 (8)	0.0094 (8)	0.0002 (8)
N4	0.0511 (11)	0.0484 (10)	0.0399 (9)	0.0044 (9)	0.0103 (8)	−0.0033 (8)
C24	0.0527 (12)	0.0368 (10)	0.0358 (9)	0.0050 (9)	0.0126 (8)	−0.0002 (8)
C25	0.0638 (15)	0.0473 (12)	0.0420 (10)	0.0069 (11)	0.0194 (10)	−0.0035 (9)
C26	0.098 (2)	0.0417 (12)	0.0532 (13)	0.0098 (13)	0.0226 (13)	0.0012 (10)
C27	0.102 (2)	0.0506 (15)	0.0646 (15)	−0.0217 (15)	0.0385 (15)	−0.0184 (12)
C28	0.0587 (14)	0.0658 (15)	0.0387 (10)	−0.0009 (12)	0.0130 (9)	−0.0165 (10)
C29	0.073 (2)	0.137 (3)	0.0522 (16)	0.011 (2)	−0.0005 (14)	−0.0383 (19)
C30	0.0681 (15)	0.0456 (12)	0.0371 (9)	0.0031 (10)	0.0164 (9)	−0.0024 (8)
C31	0.116 (3)	0.0505 (15)	0.0551 (15)	0.0064 (15)	0.0300 (16)	0.0097 (12)
C32	0.088 (2)	0.0659 (17)	0.0417 (11)	0.0049 (14)	0.0243 (12)	−0.0104 (11)

### Geometric parameters (Å, °)

**Table:**

C1—N1	1.307 (3)	C17—N3	1.297 (3)
C1—C8	1.429 (3)	C17—C24	1.435 (3)
C1—C12	1.500 (3)	C17—C28	1.511 (3)
N1—C2	1.391 (3)	N3—C18	1.395 (3)
C2—C3	1.408 (3)	C18—C19	1.409 (3)
C2—C7	1.403 (3)	C18—C23	1.405 (3)
C3—C4	1.371 (3)	C19—C20	1.373 (3)
C3—H3	0.9300	C19—H19	0.9300
C4—C5	1.392 (3)	C20—C21	1.399 (3)
C4—Cl1	1.726 (2)	C20—Cl3	1.732 (2)
C5—C6	1.372 (3)	C21—C22	1.377 (3)
C5—Cl2	1.730 (2)	C21—Cl4	1.723 (2)
C6—C7	1.413 (3)	C22—C23	1.403 (3)
C6—H6	0.9300	C22—H22	0.9300
C7—N2	1.390 (3)	C23—N4	1.398 (3)
N2—C8	1.299 (3)	N4—C24	1.297 (3)
C8—C9	1.508 (3)	C24—C25	1.503 (3)
C9—C10	1.542 (3)	C25—C26	1.536 (4)
C9—C14	1.556 (3)	C25—C30	1.551 (3)
C9—H9	0.9800	C25—H25	0.9800
C10—C11	1.533 (4)	C26—C27	1.534 (5)
C10—H10A	0.9700	C26—H26A	0.9700
C10—H10B	0.9700	C26—H26B	0.9700
C11—C12	1.552 (3)	C27—C28	1.555 (4)
C11—H11A	0.9700	C27—H27A	0.9700
C11—H11B	0.9700	C27—H27B	0.9700
C12—C13	1.512 (4)	C28—C29	1.510 (4)
C12—C14	1.571 (3)	C28—C30	1.559 (3)
C13—H13A	0.9600	C29—H29A	0.9600
C13—H13B	0.9600	C29—H29B	0.9600
C13—H13C	0.9600	C29—H29C	0.9600
C14—C15	1.516 (4)	C30—C31	1.528 (4)
C14—C16	1.549 (3)	C30—C32	1.525 (3)
C15—H15A	0.9600	C31—H31A	0.9600
C15—H15B	0.9600	C31—H31B	0.9600
C15—H15C	0.9600	C31—H31C	0.9600
C16—H16A	0.9600	C32—H32A	0.9600
C16—H16B	0.9600	C32—H32B	0.9600
C16—H16C	0.9600	C32—H32C	0.9600
			
N1—C1—C8	124.23 (19)	N3—C17—C24	124.46 (19)
N1—C1—C12	128.5 (2)	N3—C17—C28	128.6 (2)
C8—C1—C12	107.24 (18)	C24—C17—C28	106.85 (19)
C1—N1—C2	113.48 (18)	C17—N3—C18	113.25 (19)
N1—C2—C3	118.2 (2)	N3—C18—C19	118.16 (19)
N1—C2—C7	121.72 (19)	N3—C18—C23	122.47 (18)
C3—C2—C7	120.05 (19)	C19—C18—C23	119.35 (19)
C4—C3—C2	119.3 (2)	C20—C19—C18	119.8 (2)
C4—C3—H3	120.3	C20—C19—H19	120.1
C2—C3—H3	120.3	C18—C19—H19	120.1
C3—C4—C5	120.9 (2)	C19—C20—C21	120.8 (2)
C3—C4—Cl1	119.35 (19)	C19—C20—Cl3	119.33 (18)
C5—C4—Cl1	119.72 (18)	C21—C20—Cl3	119.89 (17)
C6—C5—C4	120.8 (2)	C22—C21—C20	120.2 (2)
C6—C5—Cl2	118.45 (19)	C22—C21—Cl4	119.14 (18)
C4—C5—Cl2	120.74 (18)	C20—C21—Cl4	120.62 (17)
C5—C6—C7	119.6 (2)	C21—C22—C23	119.9 (2)
C5—C6—H6	120.2	C21—C22—H22	120.1
C7—C6—H6	120.2	C23—C22—H22	120.1
N2—C7—C6	117.5 (2)	N4—C23—C22	117.9 (2)
N2—C7—C2	123.25 (19)	N4—C23—C18	122.15 (18)
C6—C7—C2	119.2 (2)	C22—C23—C18	119.9 (2)
C8—N2—C7	113.00 (19)	C24—N4—C23	113.4 (2)
N2—C8—C1	124.31 (19)	N4—C24—C17	124.2 (2)
N2—C8—C9	129.3 (2)	N4—C24—C25	129.9 (2)
C1—C8—C9	106.22 (18)	C17—C24—C25	105.84 (19)
C8—C9—C10	104.05 (18)	C24—C25—C26	103.69 (19)
C8—C9—C14	99.52 (18)	C24—C25—C30	100.70 (19)
C10—C9—C14	102.10 (19)	C26—C25—C30	102.4 (2)
C8—C9—H9	116.3	C24—C25—H25	116.0
C10—C9—H9	116.3	C26—C25—H25	116.0
C14—C9—H9	116.3	C30—C25—H25	116.0
C9—C10—C11	104.06 (19)	C27—C26—C25	103.6 (2)
C9—C10—H10A	110.9	C27—C26—H26A	111.0
C11—C10—H10A	110.9	C25—C26—H26A	111.0
C9—C10—H10B	110.9	C27—C26—H26B	111.0
C11—C10—H10B	110.9	C25—C26—H26B	111.0
H10A—C10—H10B	109.0	H26A—C26—H26B	109.0
C10—C11—C12	104.48 (18)	C26—C27—C28	104.4 (2)
C10—C11—H11A	110.9	C26—C27—H27A	110.9
C12—C11—H11A	110.9	C28—C27—H27A	110.9
C10—C11—H11B	110.9	C26—C27—H27B	110.9
C12—C11—H11B	110.9	C28—C27—H27B	110.9
H11A—C11—H11B	108.9	H27A—C27—H27B	108.9
C1—C12—C13	115.7 (2)	C29—C28—C17	115.0 (2)
C1—C12—C11	102.89 (18)	C29—C28—C30	119.7 (2)
C13—C12—C11	115.9 (2)	C17—C28—C30	99.48 (19)
C1—C12—C14	99.56 (18)	C29—C28—C27	115.7 (3)
C13—C12—C14	119.1 (2)	C17—C28—C27	103.3 (2)
C11—C12—C14	101.02 (19)	C30—C28—C27	100.9 (2)
C12—C13—H13A	109.5	C28—C29—H29A	109.5
C12—C13—H13B	109.5	C28—C29—H29B	109.5
H13A—C13—H13B	109.5	H29A—C29—H29B	109.5
C12—C13—H13C	109.5	C28—C29—H29C	109.5
H13A—C13—H13C	109.5	H29A—C29—H29C	109.5
H13B—C13—H13C	109.5	H29B—C29—H29C	109.5
C15—C14—C16	108.1 (2)	C31—C30—C32	107.8 (2)
C15—C14—C9	113.9 (2)	C31—C30—C25	112.7 (2)
C16—C14—C9	113.3 (2)	C32—C30—C25	114.0 (2)
C15—C14—C12	113.9 (2)	C31—C30—C28	114.4 (2)
C16—C14—C12	112.9 (2)	C32—C30—C28	113.4 (2)
C9—C14—C12	94.46 (17)	C25—C30—C28	94.44 (18)
C14—C15—H15A	109.5	C30—C31—H31A	109.5
C14—C15—H15B	109.5	C30—C31—H31B	109.5
H15A—C15—H15B	109.5	H31A—C31—H31B	109.5
C14—C15—H15C	109.5	C30—C31—H31C	109.5
H15A—C15—H15C	109.5	H31A—C31—H31C	109.5
H15B—C15—H15C	109.5	H31B—C31—H31C	109.5
C14—C16—H16A	109.5	C30—C32—H32A	109.5
C14—C16—H16B	109.5	C30—C32—H32B	109.5
H16A—C16—H16B	109.5	H32A—C32—H32B	109.5
C14—C16—H16C	109.5	C30—C32—H32C	109.5
H16A—C16—H16C	109.5	H32A—C32—H32C	109.5
H16B—C16—H16C	109.5	H32B—C32—H32C	109.5
